# A Multi-Omics Approach Identifies Key Hubs Associated with Cell Type-Specific Responses of Airway Epithelial Cells to Staphylococcal Alpha-Toxin

**DOI:** 10.1371/journal.pone.0122089

**Published:** 2015-03-27

**Authors:** Erik Richter, Manuela Harms, Katharina Ventz, Philipp Gierok, Ravi Kumar Chilukoti, Jan-Peter Hildebrandt, Jörg Mostertz, Falko Hochgräfe

**Affiliations:** 1 Competence Center Functional Genomics, Junior Research Group Pathoproteomics, University of Greifswald, 17489, Greifswald, Germany; 2 Department of Biochemistry, University of Greifswald, 17487, Greifswald, Germany; 3 Interfaculty Institute for Genetics and Functional Genomics, Department of Functional Genomics, University of Greifswald, 17489, Greifswald, Germany; 4 Animal Physiology and Biochemistry, Zoological Institute, University of Greifswald, 17487, Greifswald, Germany; University of Maryland, UNITED STATES

## Abstract

Responsiveness of cells to alpha-toxin (Hla) from *Staphylococcus aureus* appears to occur in a cell-type dependent manner. Here, we compare two human bronchial epithelial cell lines, i.e. Hla-susceptible 16HBE14o- and Hla-resistant S9 cells, by a quantitative multi-omics strategy for a better understanding of Hla-induced cellular programs. Phosphoproteomics revealed a substantial impact on phosphorylation-dependent signaling in both cell models and highlights alterations in signaling pathways associated with cell-cell and cell-matrix contacts as well as the actin cytoskeleton as key features of early rHla-induced effects. Along comparable changes in down-stream activity of major protein kinases significant differences between both models were found upon rHla-treatment including activation of the epidermal growth factor receptor EGFR and mitogen-activated protein kinases MAPK1/3 signaling in S9 and repression in 16HBE14o- cells. System-wide transcript and protein expression profiling indicate induction of an immediate early response in either model. In addition, EGFR and MAPK1/3-mediated changes in gene expression suggest cellular recovery and survival in S9 cells but cell death in 16HBE14o- cells. Strikingly, inhibition of the EGFR sensitized S9 cells to Hla indicating that the cellular capacity of activation of the EGFR is a major protective determinant against Hla-mediated cytotoxic effects.

## Introduction

Alpha-toxin (or alpha-hemolysin, Hla) is a major pore-forming cytotoxin released by most *Staphylococcus aureus* strains and a key factor in the pathogenesis of *S*. *aureus* diseases, including pneumonia [1–3]. The interaction of Hla with susceptible host cells is characterized by attachment to the membrane, oligomerization to a heptameric structure followed by formation of a transmembrane pore with 1–3 nm inner diameter [4–7]. Cellular responses to Hla are concentration and cell-type dependent indicating a specific mechanism by which Hla binds to the surface of host cells. Certain lipid components, particularly phosphocholine headgroups, and proteins such as caveolin-1 or disintegrin and metalloproteinase domain-containing protein 10 (ADAM10) were suggested to function as membrane receptors for Hla [8–10]. Interaction of Hla with ADAM10 may activate this metalloprotease and thereby mediate cytotoxic effects in host cells [3].

Depending on cell-type and toxin concentration, the cellular reactions to Hla-treatment are diverse, ranging from cell death to survival with defined cell-specific responses [3]. Following Hla-treatment, membranes of susceptible cells seem to become permeable for monovalent ions [11–14] and possibly also for calcium [15]. Increases of intracellular calcium levels may activate protein kinases or stimulate hydrolysis of membrane phospholipids thereby generating precursors that are central to cell signaling pathways [3, 4, 16]. In many cell types, Hla-treatment is followed by a transient decline in intracellular ATP concentrations [4, 12–15, 17, 18]. Recently, we provided evidence that a variety of intracellular metabolites, such as nucleotides and amino acids, leak out of Hla-treated viable airway epithelial cells [18].

It was demonstrated earlier that interaction of Hla with host cells can alter cell proliferation, inflammatory responses, cytokine secretion, as well as cell-cell and cell-matrix interactions [16, 19–25]. In epithelial and endothelial cells, primary disturbance of the tissue barrier function by Hla has been highlighted. Two different mechanisms are likely implicated in this process. Hla-mediated activation of the metalloprotease ADAM10 and subsequent cleavage of cadherin molecules results in loss of adherence junctions in adjacent cells [22, 23]. The Hla-ADAM10 interaction may also alter the phosphorylation states of proteins critical in the regulation of the dynamics of cell-basement membrane contacts (focal adhesions) leading to their dissolution [9]. Furthermore, the Hla-mediated secretion of pro-inflammatory cytokines and chemokines from airway epithelial cells has previously been shown to be mediated via activation of ERK-type and p38 MAP kinases [16, 26].

Although protein phosphorylation-mediated signaling in airway epithelial cells seems to be critical for cellular responses towards Hla, alterations have not been investigated thoroughly so far. Here, we utilized phosphoproteomics in 16HBE14o- and S9 human bronchial epithelial cells in order to highlight critical pathways affected by Hla-treatment. We validate activity profiles of many identified kinases and down-stream substrates by Western blot analyses and correlate differentially activated kinases in both cell systems to the observed differences in Hla-mediated cytotoxicity. Additionally, we describe early Hla-associated alterations in protein expression levels by transcriptomic and proteomic approaches.

## Materials and Methods

### Recombinant α-toxin

Recombinant Hla (rHla) from *S*. *aureus* was expressed and purified as described previously [26]. The purity was evaluated with a Coomassie-stained SDS-gel and hemolysis activity was tested on blood agar plates. For controls, a mock purification from *Escherichia coli* containing vector DNA only was carried out.

### Cell culture and SILAC

The two immortalized human airway epithelial cell lines 16HBE14o- and S9 are frequently used as model cells for studying cellular functions of human airways [27–30]. S9 cells were originally derived from a cystic fibrosis patient, subsequently corrected by introduction of the gene encoding wild-type cystic fibrosis transmembrane conductance regulator (CFTR) through adenoviral transfer. 16HBE14o- cells were derived from the bronchial epithelium of a transplant patient and express wild-type CFTR. The 16HBE14o- cells are highly polarized and all known characteristics indicate that these cells represent one type of serous cells at the surface of airway epithelia, whereas the less polarized S9 cells may represent an airway cell type that is derived from the lower layer of the bronchial epithelium [27, 30, 31].

16HBE14o- and S9 cells were cultivated at 37°C with 5% CO_2_ in a humidified atmosphere in RPMI-1640 (with L-glutamine and sodium bicarbonate, without arginine, leucine, lysine; Sigma-Aldrich) supplemented with 382 μM L-leucine (Sigma-Aldrich), 218 μM L-lysine, 144/72 μM L-arginine (16HBE14o-/S9 cells), 10% dialyzed fetal bovine serum (FBS, Sigma-Aldrich) and penicillin 100 U/ml, streptomycin 100 μg/ml (Biochrom AG). For 16HBE14o- cells, growth medium was additionally supplemented with 2 mM L-glutamine (Biochrom AG). For stable isotope labelling with amino acids in cell culture (SILAC), cells were grown for at least 6 cell divisions in medium supplemented with the heavy isotope labelled forms of L-lysine and L-arginine (^13^C_6_^15^N_2_-L-lysine and ^13^C_6_^15^N_4_-L-arginine, Silantes). Medium was changed every three days. Cells were sub-cultivated routinely twice a week using EDTA (0.05% (w/v) in phosphate buffered saline (PBS), Sigma-Aldrich) and trypsin / EDTA (0.05% (m/v) / 0.02% (m/v) in PBS, Biochrom AG). Cells were detached by gently tapping and suspended in growth medium. An aliquot of the cell suspension obtained was mixed with trypan blue solution (Sigma-Aldrich) for checking cell viability and counted using a Buerker haemocytometer. All cultures were tested for absence of mycoplasma contamination by PCR on a regular basis.

For proteomic and transcriptomic experiments cells were seeded in 150 mm cell culture dishes at a density of 5.5 x 10^6^ cells. Confluent cell layers were treated with 2,000 ng/ml rHla or an equivalent volume of mock control for 2 hours. SILAC-experiments were carried out in duplicate with switched labels. Transcriptomic experiments were performed in biological duplicates.

### Cell viability and survival assays

Cells were seeded in 24-well plates at a density of 0.2 x 10^6^ cells. After reaching confluency, cells were treated with 2,000 ng/ml rHla or mock control. After 2, 6 or 24 hours cell viability was determined by cell counting as described above. Experiments were carried out at least in triplicate with three parallels each. For analysis of Hla cytotoxicity cells were seeded in 96-well plates at a density of 0.5 x 10^4^ cells (for both, 16HBE14o- and S9 cells) for early time points, or 1 x 10^5^ and 1.5 x10^5^ cells for long term analysis for 16HBE14o- and S9 cells, respectively. Cells were treated with 2,000 ng/mL of rHla for the indicated time points. The general metabolic condition of cells was determined by resazurin staining (Sigma-Aldrich) and absorbance was measured at 570 nm and 600 nm using a multiwell plate reader (Synergy Mx, BioTek). Experiments were carried out in triplicate with 6 parallels each.

### Protein kinase inhibitors

Cells were seeded in 24-well plates at a density of 0.2 x 10^6^ cells and treated with 10 μM tyrphostin AG1478 (Sigma-Aldrich), PD98059 (Cell Signaling Technology) or vehicle 1 h prior to addition of rHla. Cell viability was determined 6 h post rHla-treatment by cell counting. Experiments were carried out in triplicates with three parallels.

### Preparation of samples for proteome and phosphoproteome profilings

Cells were harvested by directly scraping in lysis buffer (8 M urea, 10 mM sodium fluoride, 2.5 mM sodium pyrophosphate, 1 mM β-glycerol phosphate, 1 mM sodium orthovanadate, 1 mM tris (2-carboxyethyl) phosphine (TCEP), 1 mM EDTA, and 20 mM HEPES, pH 8.0) and immediately frozen. Lysed cells were thawed, sonicated and free protein thiols were alkylated by addition of iodoacetamide to a final concentration of 5 mM followed by incubation for 20 min at room temperature. Lysates were clarified by centrifugation (8,700 x g, 15 min) and protein content was quantified by the Bradford method (Bio-Rad). Equal amounts of differentially labelled proteins were mixed for further sample processing. For protein expression profiling, 20 μg protein mix was fractionated into 10 gel slices by one-dimensional SDS-PAGE. Peptides were obtained by in-gel digestion using trypsin (Promega). For enrichment of phosphopeptides, 5 mg (for TiO_2_-based enrichment) or 20 mg (for immuno-based enrichment) of protein mixtures were diluted eightfold with 20 mM HEPES, pH 8.0 and digested with trypsin using a protein-enzyme ratio of 20:1. Obtained peptides were purified using C18 Sep-Pak cartridges (Waters) and freeze-dried. Peptides destined for TiO_2_-based enrichment were dissolved in buffer A (5 mM KH_2_PO_4_, 30% (v/v) acetonitrile, pH 2.7) and separated in 15 fractions by strong ion exchange chromatography (SCX) with a ResourceS SCX column (1 ml column volume, GE) connected to an Äkta Avant chromatography system (GE) using a binary linear gradient with buffer B (5 mM KH2PO4, 350 mM KCl, 30% (v/v) acetonitrile, pH 2.7) at a flow rate of 1 ml/min. Fractions were lyophilized, dissolved in 14 ml TiO_2_-binding buffer (73% (v/v) acetonitrile, 10% (v/v) lactic acid, 2% (v/v) TFA) and 100 μl from a TiO_2_-stock solution (30 mg / ml Titansphere TiO_2_ bulk material (GL sciences) in 100% acetonitrile) was added, followed by incubation for 20 min at room temperature. Samples were centrifuged, beads were washed four times with 80% (v/v) acetonitrile, 2% (v/v) TFA and phosphopeptides were sequentially eluted with 5% (v/v) NH4OH and 30% (v/v) acetonitrile. Samples for enrichment of tyrosine-phosphorylated peptides by immunoprecipitation were dissolved in IP buffer (50 mM MOPS, 10 mM Na2HPO4, 130 mM NaCl, 0.5% (v/v) NP40, pH 7.5) and incubated overnight at 4°C with P-Tyr-100 beads prepared by coupling of 300 μg of a monoclonal mouse anti-phosphotyrosine antibody (P-Tyr-100, Cell Signaling Technology) to rec-Protein G-Sepharose 4B beads (Life Technologies) in a 1:4 ratio. Beads were washed three times with IP buffer and twice with water. Tyrosine-phosphorylated peptides were eluted twice with 50 μl 0.1% (v/v) TFA and once with 15% (v/v) acetonitrile, 0.1% (v/v) TFA. Eluates were vacuum-dried and purified with C18 Stage-Tips (Thermo Scientific). Peptides were again vacuum-dried and stored at -20°C.

### Mass spectrometry and spectra analysis

LC-MS/MS analyses were performed using an EASY-nLCII nanoflow HPLC system coupled directly to an LTQ Orbitrap Velos hybrid mass spectrometer (Thermo Fisher Scientific). Peptide samples were dissolved in 20 μl 5% (v/v) acetonitrile, 0.1% (v/v) acetic acid and loaded onto a 20 cm-long self-packed C18 (Aeris Peptide 3.6 μm, pore size 100 Å; Phenomenex) analytical column. Gradual elution of peptides was achieved by running a binary linear gradient from 1% (v/v) acetonitrile/0.1% (v/v) acetic acid to 75% (v/v) acetonitrile/0.1% (v/v) acetic acid over a period of 46 minutes (TiO_2_ samples) or 80 minutes (pTyr-IP samples) with a flow rate of 300 nl/min. The MS was operated in data-dependent mode, automatically switching between full survey scan (m/z 300–1700) with a resolution of 30,000 followed by fragmentation analyses of the top 10 precursors with a charge state greater than one. MS/MS analyses were either with collision induced dissociation (CID) or higher-energy collisional dissociation (HCD). Fragmentation by CID was performed in the linear ion trap with an AGC target value of 5 x 10^3^ ions and normalized collision energy of 35%. Spectra for HCD fragmentation were acquired in the Orbitrap mass analyzer with a target value of 5 x 10^4^ ions and 40% normalized collision energy. Precursors were dynamically excluded for repeated fragmentation for 30 s and 20 s for the CID and HCD method, respectively.

Obtained raw data files were processed using MaxQuant (vers. 1.2.2.5) with the integrated Andromeda search engine [32, 33] against the reviewed human proteome deposited in UniProtKB (Swiss-Prot database vers. 2011_11; released date: 2011-11-16; 20,251 sequences). Trypsin was specified as enzyme and a total number of 2 missed cleavages were allowed. Arg10 and Lys8 were set as heavy labels. Carbamidomethylation of cysteine residues was set as fixed modification. Oxidation of methionine, amino-terminal acetylation and phosphorylation of serine, threonine and tyrosine were selected as variable modifications. The ‘requantify’ and ‘match between runs’ options were enabled. For peptide, protein or site-specific identifications a false discovery rate cut-off of 0.01 was applied. Provided SILAC ratios are based on average values. Only SILAC ratios calculated from at least two independent events were taken into account. For protein expression analysis, only SILAC ratios from protein groups with at least two independent peptide identifications, including at least one unique peptide, were considered.

### Immunoblotting

Primary antibodies used in this study were anti-ADAM10 (abcam #ab1997), PE conjugated anti-ADAM10 (Biolegend #352704), anti-v-Src (Calbiochem #OP07), anti-Src family pY416 (CST #2101), anti-MAPK14 (CST #9212, anti-MAPK14 pT180/pY182 (CST #9211), anti-FAK (BD #610088), anti-FAK pY397 (Biosource #44-625G), anti-FAK pY576 (Santa Cruz #sc-16563), anti-FAK pS910 (Biosource #44-596G), anti-MAPK3/1 (CST #9102), anti-MAPK3/1 pT202/pY204 (CST #9106), anti-PAK2 (CST #2608), anti-PAK1/2 pS144/pS141 (CST #2606), anti-BCAR1 (Santa Cruz #sc-860), anti-BCAR1 pY249 (CST #4014), anti-Paxillin (Santa Cruz #sc-5574), anti-Paxillin pY118 (CST #2541), anti-Shc pY239/pY240 (CST # 2434), anti-Gab1 pY627 (CST #3231), PE-conjugated anti-E-cadherin (BioLegend #324106), anti-EGFR (Biolegend #352904), anti-Vinculin (Life Technologies #700062).

Protein extracts were separated by one-dimensional SDS gel electrophoresis using 4–15% TGX gradient gels (Biorad) and subsequently transferred on a PVDF membrane (Merck-Millipore). Membranes were blocked for 1.5 h in 5% (w/v) dried milk in PBS-0.05% (v/v) Tween-20 and probed with various phospho-site specific primary antibodies and their corresponding pan antibodies overnight at 4°C. Detection was performed using either IRDye680RD or IRDye800CW secondary antibodies (LI-COR) and the Odyssey infrared imaging system (LI-COR).

### Microscopy

For fluorescence microscopic analysis 1 x 10^4^ cells were seeded onto coverslips. After rHla or mock treatment for 2 h cells were washed with DPBS and fixed with 4% (w/v) paraformaldehyde (Sigma) for 10 minutes at room temperature, permeabilized in 0.2% (v/v) Triton X-100 (Sigma) for 5 min on ice and blocked with 1% (w/v) BSA in DPBS for 10 minutes. Cells were stained for 30 min with phycoerythrin-conjugated anti-E-cadherin in DPBS containing 1% (w/v) BSA, washed twice with BSA-DPBS and sealed with fluoromount (Sigma) or stained for 20 min with an anti-Vinculin antibody (Life Technologies) in BSA-DPBS followed by probing with an AlexaFluor488- conjugated secondary antibody (Life Technologies), rhodamine phalloidin (Life Technologies) and Hoechst 33342 (Sigma) staining for 45 min. Coverslips were sealed with fluoromount and fluorescence microscopy was performed using a Zeiss Axio Observer.

### Gene expression analysis

Total RNA was isolated using the TRIzol reagent (Invitrogen). RNA was purified using the RNA Clean-Up and Concentration Micro Kit (Norgen) and concentrations were measured using a ND-1000 spectrophotometer (Thermo Fisher Scientific Inc). RNA integrity was validated by means of the lab-on-chip capillary electrophoresis technology (Bioanalyzer 2100, Agilent Technologies). Only RNA samples with an RNA integrity number (RIN)>9.5 [34], 260/280 nm≥1.8, 260/230 nm≥1.9 were used for microarray analyses. For each sample, 200 ng of total RNA was reverse transcribed into cDNA, amplified, and in vitro transcribed to cRNA. Sense-strand cDNA was generated from 10 μg of purified cRNA using random primers, followed by fragmentation and labelling using 5.5 μg of purified sense-strand DNA. Biotinylated sense-strand DNA was then hybridized onto the Affymetrix GeneChip Human Gene 1.0 ST arrays for 16 h. Arrays were washed and stained using the Fluidics Station 450. Scanning was performed by GeneChip Scanner 3000 7G (Affymetrix); raw CEL files were generated using the GCOS software. Quality assessment of all hybridizations was carried out by inspecting scan images and by reviewing external and endogenous controls using the Expression Console software (Affymetrix).

Data analysis was carried out using Rosetta Resolver system for gene expression data analysis (Rosetta Bio software). In brief, the raw signals of the gene-specific probes were summarized using the Robust Multi-array Average algorithm and data transformation for array comparability was achieved by performing quantile normalization. Genes exhibiting significantly different expression on the RNA level were identified using the following cut-off criteria: one-way analysis of variance with subsequent Benjamini and Hochberg false discovery rate multiple-testing correction on pair-wise comparisons (ANOVA, p≤0.05), signal correction statistics (Ratio Builder, p≤0.05) and fold-change≥1.5-fold. Probe-set transformation into genes was performed by using the Rosetta Resolver transformation tool based on the Entrez Genes/Unigenes search engine (NCBI).

### Functional classification, downstream effects and upstream regulator analysis

For protein and phosphoprotein data, functional annotation enrichment was based on KEGG pathways (DAVID Bioinformatics Resources 6.7; [35]. Kinase-substrate relationships and protein-protein interactions were downloaded from the PhosphoSitePlus (www.phosphosite.org; [36]) and STRING database [37] and visualized with Cytoscape version 2.8.3 [38] and Adobe Illustrator CS6.

Functional trends caused by the change of gene expression were assessed using IPA Down Stream Effects Analysis (Ingenuity Systems). In brief, protein IDs or transcript-specific probe sets corresponding to differentially expressed genes were tabulated and IPA core analysis was performed. Top hits from the Molecular and Cellular Functions category were then extracted based on Fisher’s exact test (p-value≤0.01, Benjamini-Hochberg multiple testing corrected) as an estimation of the probability of the association between groups of genes and cellular functions due to random chance. IPA regulation z-score greater than 2 (increased activation) or lower -2 (decreased activation) was used to predict whether observed cellular functions are activated or deactivated after rHla-treatment. Identification of upstream regulators was achieved through the correlation with differentially expressed genes by the use of IPA Upstream regulator analysis (Ingenuity Systems). Upstream regulators of the molecule type growth factor, kinase, and transmembrane receptor were assumed as valid effectors of gene expression after rHla-treatment if the corresponding p-value obtained by Fisher’s exact test was equal to or less than 0.01. Activation z-score algorithm was used to allow for prediction whether an upstream regulator is activated (z≥2) or inactivated (z≤-2) based on the direction of expressional change of the associated genes.

### Flow cytometry

Flow cytometry was used to determine the surface expression of ADAM10, EGFR and E-cadherin on 16HBE14o- and S9- cells. For flow cytometric analysis, cells were trypsinized and 1 x 10^6^ cells were stained with PE-conjugated anti-ADAM10 (BioLegend), anti-E-cadherin (BioLegend), anti-EGFR (BioLegend) or appropriate isotype control (IgG1k, eBioscience) antibodies in 100 μl medium for 30 min at 4°C in the dark. Cells were washed twice with FACS buffer (DPBS, 1% (v/v) FBS, 3.8 mM sodium azide) and resuspended therein. Stained cells were analyzed on an Attune Acoustic Focusing Cytometer (Life Technologies). Ten thousand events were gated and analyzed with Attune software V2.1.0 or FlowJo V10.07 (Tree Star).

### ADAM10 knockdown

For ADAM10 knockdown in 16HBE14o- and S9 cells, siRNAs HS_ADAM10_4 (target sequence ATG GTG TTG CTG AGA CTG TTA), HS_ADAM10_5 (target sequence TTG GTG GGC AGT ATT ACT TAT) or negative control (Qiagen) were used according to the manufacturer`s protocol. Cells were seeded in 96-well plates at a density of 0.5 x 10^4^ cells or in 60 mm plates at a density of 0.5 x 10^6^ cells. 24 h after seeding, cells were transfected with siRNAs using Lipofectamine for 24 h. Medium was exchanged and cells were left undisturbed for additional 48 h.

## Results

### Staphylococcal alpha-toxin decreases viability in 16HBE14o- but not in S9 cells

In previous studies, different rHla concentrations were tested on 16HBE14o- and S9 epithelial cells. The dose-response relationship for rHla-mediated cyto-/chemokine release thereby showed highest values for 2,000 ng/ml rHla [16]. Furthermore, retardation of cell growth for 16HBE14o- cells was unaffected by up to 200 ng/ml rHla as determined by impedance measurements and most pronounced when cells were treated with 2,000 ng/ml [25]. In addition, microscopic inspection revealed that this concentration resulted in the liberation of cells from the layer and irreversible formation of paracellular gaps for 16HBE14o-, A549 and primary human epithelial cells isolated from nasal polyps in the long term but this effect was only moderate and transient in S9 cells [25]. For the characterization of rHla-mediated effects on the metabolome [18], transcriptome and (phospo-)proteome, we adopted the concentration of 2,000 ng/ml for our omic studies by carefully testing cell survival of confluent cell layers of S9 and 16HBE14o- human bronchial epithelial cells under our experimental conditions.

**Fig 1:**
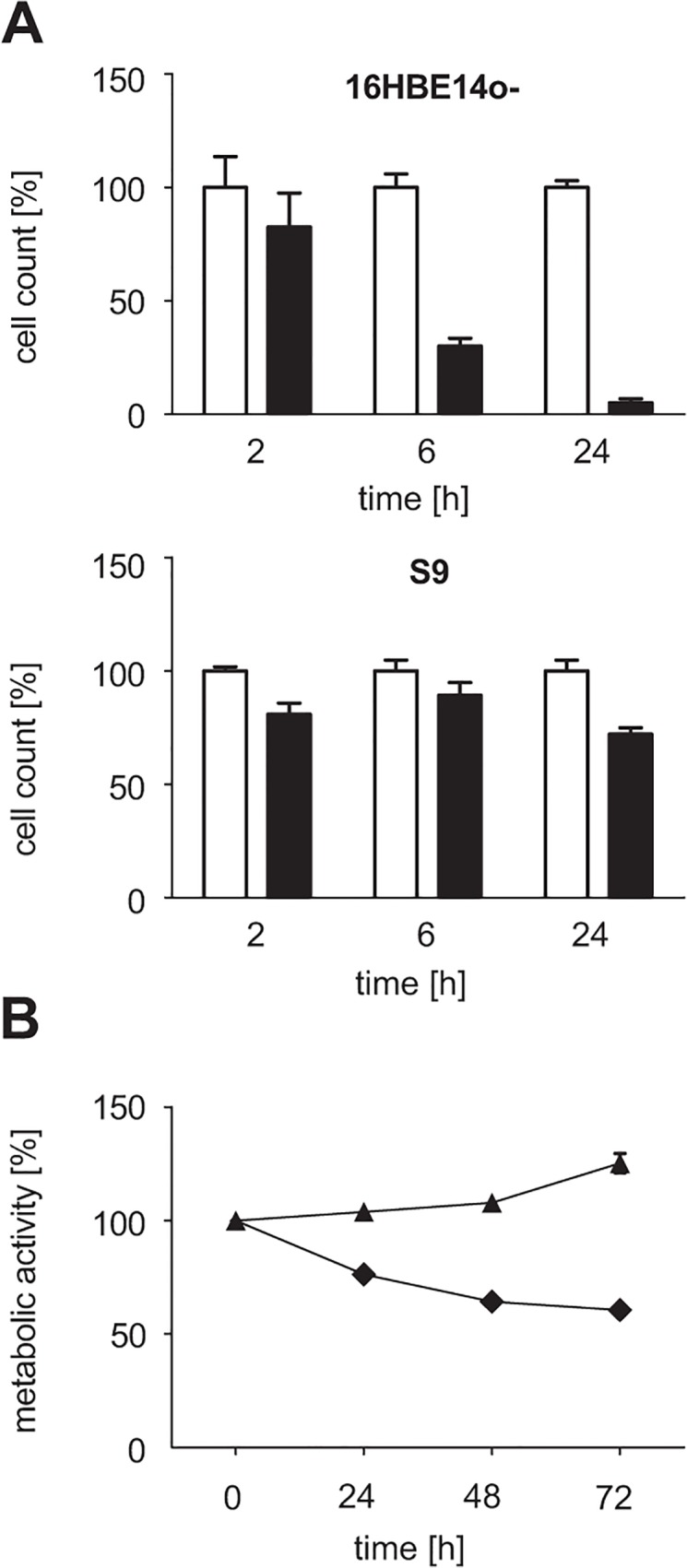
Cell survival and general metabolic fitness of the human bronchial epithelial cells 16HBE14o- and S9 after treatment with 2,000 ng/ml rHla. A. Cell counts of 16HBE14o- and S9 cells 2 h, 6 h and 24 h post rHla-treatment (black bars) compared to mock treated cells (white bars). Data represent mean ± SEM (n = 3). B. General metabolic condition of rHla-treated 16HBE14o- (diamonds) and S9 (triangles) cells as indicated by a resazurin-based assay. Measurements after rHla-treatment are normalized to the initial reading before rHla addition. Data represent mean ± SEM (n = 3).

As shown in Fig. 1A, proportions of viable 16HBE14o- cells were 80%, 30% and 5% of the respective controls after incubation for 2, 6 and 24 h, respectively. In contrast, only decreases of less than 30% in viable cells were observed for S9 cells within the same periods. We also determined rHla-mediated effects on the general metabolic condition of the cells using a Resazurin-based assay. Corresponding to the cell type-specific changes in cell numbers, a rHla-induced drop in fitness was observed from 90% at 10 min to 50% at 24 h for 16HBE14o- cells, whereas S9 cells were much less affected by rHla (less than 9%) over the same period of time.

To test if surviving S9 cells recover from rHla-treatment, sub-confluent cell cultures were assayed with the Resazurin assay over a period of 72 h (Fig. 1B). As expected, values for 16HBE14o- cells declined. However, S9 cells showed an increase in Resazurin conversion rates for subsequent sampling points indicating that they are capable of overcoming Hla-mediated cytotoxicity.

In summary, in agreement with previous studies 2,000 ng/ml rHla is cytotoxic to 16HBE14o- but not to S9 cells under the experimental conditions used. As the aim of our work was to study early Hla-mediated cellular responses associated with cellular signaling and gene expression, we decided on a time point of two hours after rHla addition for further system-wide quantitative analyses. At this time point, rHla-mediated cellular signaling processes were expected to be fully activated, but cytotoxic effects of rHla were not yet eminent.

### Alpha-toxin induces substantial changes in the phosphoproteomes of 16HBE14o- and S9 cells

**Fig 2:**
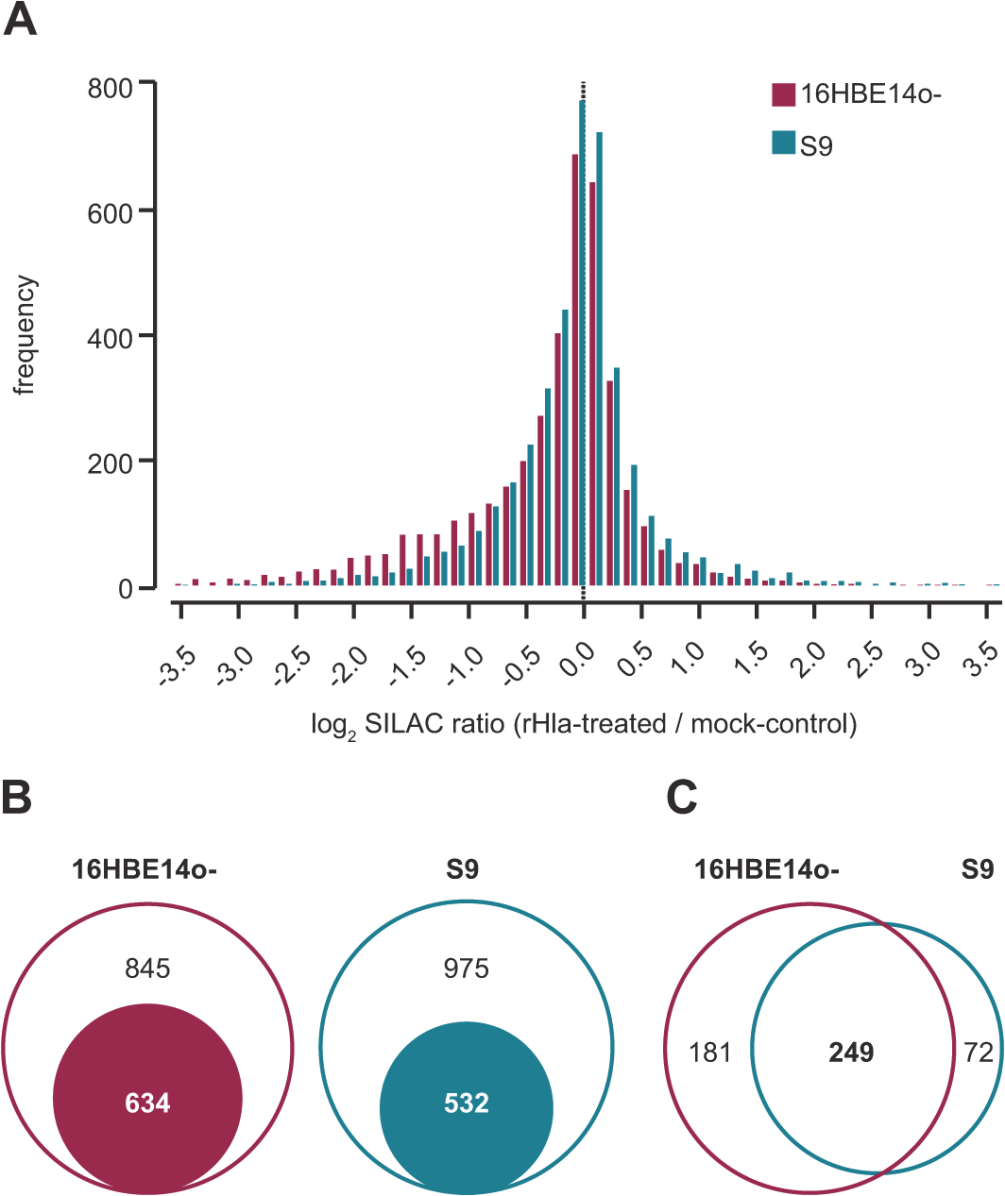
Comparative overview about the phosphoproteomic data of 16HBE14o- and S9 cells treated with rHla for two hours. A. Histogram depicting the distribution of SILAC phosphopeptide ratios of Hla- vs. mock-treated cells. B. Euler diagram illustrating the set of proteins assigned with significant alterations in their phosphorylation status (filled circle) in relation to all phosphoproteins. C. Venn diagram showing the relation of altered phosphorylations common to both and unique to either cell types.

To elucidate alterations in phosphorylation-dependent intracellular signaling pathways associated with the action of alpha-toxin, we utilized metabolic protein labelling (SILAC, [39, 40]), phosphopeptide enrichment techniques and high-accuracy mass spectrometric (MS) characterization combining high-low and high-high strategies [41–44]. Using this workflow, we were able to quantify 5,432 modification-specific peptide sequences (based on at least two independent SILAC ratio counts) corresponding to 1,827 proteins (Fig. 2A, S1 Table). For a total of 4,523 phosphorylations (4,036 phosphoserine, 321 phosphothreonine and 167 phosphotyrosines), the modification site within the amino acid sequence of the protein was thereby mapped with high confidence (Class-I hits: Localization Probability > 75%, Score Difference > 5; S1 Table).

Considering the third quartile (Q_0.75_) of all unsigned log_2_-transformed SILAC phosphopeptide ratios from both cell lines as cut-off (1.58-fold on linear scale), 43% and 35% of the derived phosphoproteomes are affected in 16HBE14o- and S9 cells following rHla-treatment, respectively (Fig. 2B). From the 1106 proteins that are derived from overlapping phosphopeptide quantitation of both models, the half showed alterations in either system and most of them (approximately 80%) were commonly up- or down-regulated (Fig. 2C). Interestingly, most of the cell type-specific altered proteins were less phosphorylated in 16HBE14o- cells and more phosphorylated in S9 cells.

### Alpha-toxin affects major signaling pathways of cell-cell and cell-matrix contacts as well as the actin cytoskeleton

**Fig 3:**
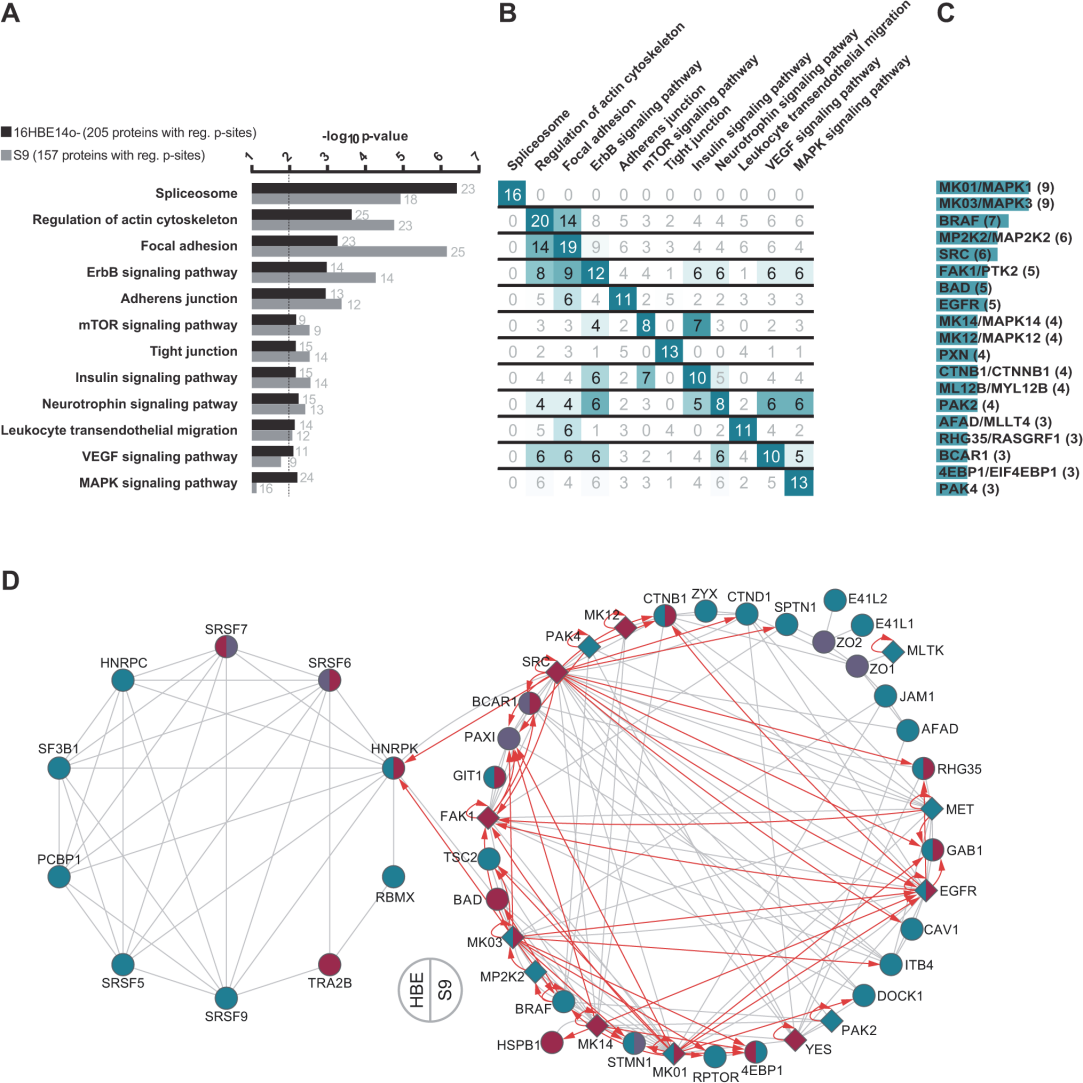
Functional annotation analysis of proteins with altered phosphorylation in rHla-treated 16HBE14o- and S9 cells. A. KEGG pathways with statistically noticeable number of proteins with altered phosphorylation following rHla-treatment for 2 h. The dotted vertical line indicates the cut-off for significant enrichment (Benjamini-Hochberg corrected p-value ≤0.01). Numbers next to the horizontal bars provide the number of assigned proteins. B. Matrix for cross-comparison of recurrent proteins with altered phosphorylation in both cell types between KEGG pathways from panel A. Numbers greater than the half of the maximum possible are highlighted. C. Ranking of phosphoproteins with altered phosphorylation that appear in at least three enriched KEGG pathways. D. Network visualization of proteins from the enriched KEGG pathways that have been identified with altered phosphorylation in both cell lines. Grey lines indicate experimentally validated protein-protein-interactions and red arrows kinase-substrate relationship taken from STRING and PhosphoSitePlus, respectively. rHla-induced changes are indicated in ruby (increased p-sites); cyan (decreased) or purple (both) for kinases (diamond shape) and substrates (circles) for 16HBE14o- cells (left half of shape) and S9 cells (right).

To identify general and cell type-specific effects on the phosphoproteome of S9 and 16HBE14o- cells by Hla, we assessed the data sets for overrepresented biological pathways and processes. Strikingly, proteomic enrichment analysis with the Kyoto Encyclopedia of Genes and Genomes (KEGG) pathway resulted in identical hits for major pathways for both cell lines (Fig. 3A). Top pathways that were significantly enriched in regulated phosphoproteins in both cell types include the spliceosome, actin cytoskeleton signaling, focal adhesion, as well as ErbB/EGFR signaling and cell-cell contact signaling.

The comparison of the individual regulated proteins across all affected pathways revealed a unique set of altered phosphoproteins associated with the spliceosome and a high degree of overlap of the remaining phosphoproteins across the other enriched pathways (Fig. 3B). In order to highlight key players, we ranked the proteins that were affected by rHla-treatment at their level of phosphorylation according to their frequency of occurrence in different pathways. In total, 15 proteins were found to be present in at least three different KEGG pathways (Fig. 3C). Remarkably, among them are 10 protein kinases—MAPK1 (ERK2), MAPK3 (ERK1), MAP2K2 (MEK2), SRC, FAK, EGFR, MAPK14 (p38 alpha), MAPK12 (p38 gamma), PAK2 and PAK4.

For selected kinases and down-stream targets, we performed Western blot analyses with cell extracts harvested 0.5, 2 and 6 hours after addition of rHla or mock treatment. Phosphosite-specific and corresponding pan antibodies for SRC, FAK1, MAPK1/3, BCAR1 and PAK2 were thereby utilized to validate the identified up- or down-regulation based on MS-SILAC ratios (S1 Fig.). As expected, the Western blot results of these key signal transduction proteins correlated well with those detected after 2 h of rHla challenge based on the MS-based phospho-profiling. The corresponding total protein levels remained largely unchanged over the monitored 6 h time period indicating direct modulation of the phosphorylation events by up-stream regulators.

Within the significantly enriched KEGG pathways, 65 proteins were phospho-regulated by rHla-mediated action in both cell lines. These proteins were subjected to interaction analyses based on known protein-protein and kinase-substrate relations. In Fig. 3D, the result is visualized in a two-circuit network separating spliceosome-associated proteins (left circle; 10 proteins) from the remaining (38 proteins). Both sub-networks show a high degree of intrinsic interconnectedness based on protein-protein interactions. Strikingly, the non-spliceosome sub-network contains 12 protein kinases, most of them with multiple and partly overlapping target proteins in the same network. The results of the pathway and network analyses suggested that Hla had a major impact on proteins that have key signaling functions in cell contact and cell anchorage processes. We therefore also used fluorescence microscopy to monitor actin, vinculin and E-cadherin as surrogate markers for morphological changes of these cell features. After 2 h of rHla-treatment, we observed a loss of actin stress fibers which was more pronounced in 16HBE14o- cells compared with S9 cells (S2B Fig.), redistribution of actin to the cellular periphery and a loss of dot-like distribution of vinculin in the cell periphery (S2B Fig.) as well as a partial loss in E-cadherin from the cell membranes (S2C Fig.) indicating down-regulation of cell-cell- or cell-matrix contacts, respectively.

### Alpha-toxin induces divergent phosphorylation patterns in key signaling proteins in S9 and 16HBE14o- cells

From the signaling pathway analyses and the phenotypical observations, we hypothesized that variations in the rHla-induced activity of individual signaling nodes between both cell models might exist that are associated with the different sensitivity towards Hla. We therefore especially screened for proteins with differential phosphorylation patterns in both models.

We ranked the proteins according to the largest difference between oppositely altered phosphopeptides. Thereby, 14 phosphoproteins could be highlighted and the mapped phosphorylation sites were assigned. Most of them, i.e. 11 proteins, showed decreased phosphorylation in 16HBE14o- cells and increased phosphorylation in S9 cells (GAB1, EGFR, EPHA2, NU153, MAPK3, MAPK1, CTNB1, TOM1, CHD1, VIGLN, LASP1) and only 3 proteins (IF4G3, PSIP1, ODPA) were found with a reciprocal phosphorylation behavior. Notably, several of the proteins with differing phosphorylation including the receptor tyrosine kinase EGFR and the cytoplasmic serine/threonine kinases MAPK3 and MAPK1 are associated with signaling pathways regulating cell contacts and the actin cytoskeleton (Fig. 3D).

The kinases EGFR, MAPK3 and MAPK1 were found to be differentially regulated at sites directly involved in the induction of down-stream kinase activity. For pT202/pY204 in MAPK3/MAPK1, increased phosphorylation in S9 and decreased phosphorylation in 16HBE14o- cells were confirmed by Western blotting (S1 Fig.). Since we failed to detect signals with phosphosite-specific antibodies in EGFR, we used the known down-stream target sites Y239 in SHC and Y627 in GAB1 as surrogate markers for validation of potentially altered EGFR activity. Following rHla-treatment, SHC-Y239 and GAB1-Y627 were found to be markedly increased in phosphorylation in S9 cells, whereas at least phosphorylation of SHC-Y239 was strongly decreased in 16HBE14o- cells (S1 Fig.) indicating increased EGFR activity in S9 and decreased activity in 16HBE14o- cells as predicted by the MS results (S1 Table).

Finally, we extended the evaluation to all 71 protein kinases detected with altered phosphorylation levels following exposure of cells to rHla. Focusing on mapped sites that are known to directly modulate kinase activity, we detected up-regulation of the mitogen-activated protein kinase 14 (p38 alpha), the focal adhesion kinase FAK and Src-family kinases common to S9 and 16HBE14o- cells (S1 Table and S1 Fig.). In contrast, the receptor tyrosine kinases MET and EphA2 were found to be strongly de-phosphorylated at activation sites indicative for reduced kinase activity following rHla exposure (S1 Table).

### Alpha-toxin causes distinctive expression changes in signaling modules relevant for cell viability and apoptotic cell death

Phosphorylation-mediated signaling more often than not involves modulation of gene expression. Therefore, we analyzed 16HBE14o- and S9 cells at the level of protein and mRNA expression under control conditions and 2 h after rHla-treatment. Examining our proteome data, we identified 3,579 different proteins based on at least two individual peptide identifications with at least one peptide unique per protein (false discovery rate ≤0.01). Within this set of proteins, 3,168 fulfill our criteria for quantification. Of note, protein kinases (e.g. EGFR, MAPK3, MAPK1, YES, FAK) and phosphatases (e.g. PTN23, ILKAP, DUS23, PGAM5, PP4C) as well as transcriptional and translational regulators (e.g. ATRX, CTCF, SMAD2, PURA/B) were included in this set indicating a sufficient depth of the analysis to cover even the expression of regulatory proteins, which are in general at the bottom of protein abundance. The application of the phosphoproteome derived cut-off values of more than 1.58-fold and less than -1.58-fold revealed moderate up- and down-regulation in protein expression following 2 h of rHla challenge. In summary, we detected 29 or 26 proteins, respectively, that appeared to be significantly increased or decreased in 16HBE14o- cells (S2 Table). For S9 cells, 33 proteins were observed at higher expression levels upon rHla-treatment of cells while 37 proteins were found in lower amounts. Glycosyltransferase glycogenin GYG-1, transmembrane 4 superfamily member TSN11, syndecan SYND4 and centromere protein F showed overlapping regulation at their protein levels in both cell lines. Interestingly, for both S9 and 16HBE14o- cells, functional category analysis of the proteins with altered abundances revealed an association with cellular growth and proliferation as well as cellular movement by trend.

**Fig 4:**
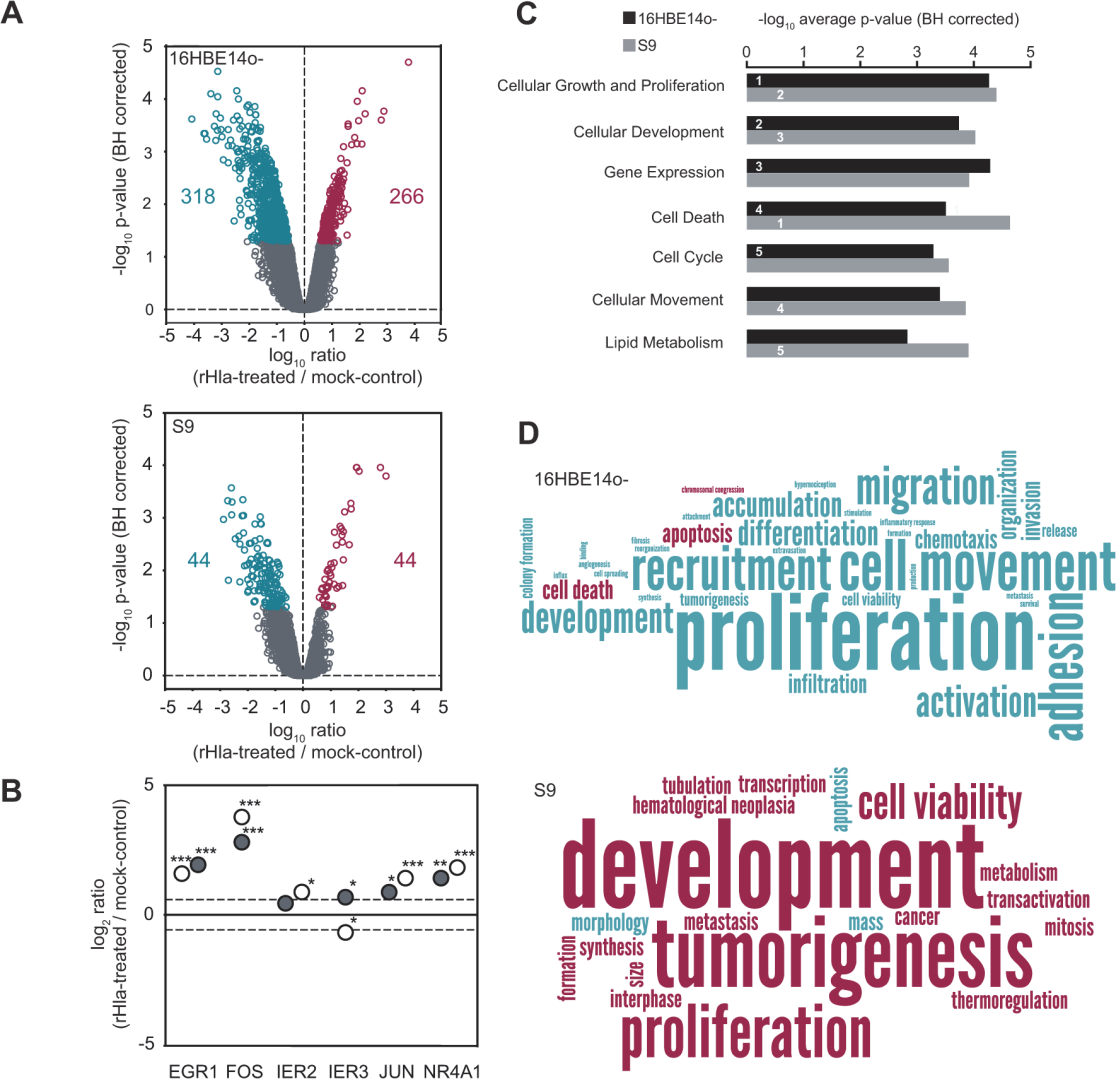
DNA-microarray-based transcription profiles of 16HBE14o- and S9 cells under influence of rHla. A. Volcano plots of mRNA expression differences under control conditions and after treatment with rHla in 16HBE14o- and S9 as a function of statistical significance (ANOVA post hoc p≤0.05) and intensity ratio at the level of probe-sets. Probe sets indicating significant higher expression after rHla-treatment are indicated in ruby, probe sets indicating higher expression under control conditions are in cyan, and probe sets with no significant expression differences are in gray. Numbers indicate the number of gene-specific mRNAs increased (ruby) and decreased (cyan) after assigning probe-sets to genes. B. Expression level changes for selected immediate early response genes after rHla treatment of 16HBE14o- (open circles) and S9 (filled circles) cells (***p<0.001; **p<0.01; *p<0.05). C. Top five cellular functions affected after rHla-treatment in the category molecular functions for 16HBE14o- (black bars) and S9 (gray bars) as predicted by IPA Downstream Effects Analysis based on differentially expressed genes. D. Functional trends after rHla-treatment in all categories as predicted by IPA Downstream Effects Analysis based on direction of change of differentially expressed genes (16HBE14o- upper panel, S9 lower panel). The word cloud depicts the frequency of terms by font size and predicted increase in functional activity is shown in ruby and decrease in activity is shown in cyan.

Hence, we asked if the observed moderate changes in the proteome following rHla-treatment are preceded or accompanied by alterations at the transcriptional level. For this purpose, global transcriptome analyses of rHla-treated S9 and 16HBE14o- cells were performed. Two hours after the addition of rHla, we monitored 205 (S9) and 915 (16HBE14o-) gene-specific probe sets pointing to their corresponding genes with differential expression (Fig. 4A, S3 Table). Probe-set transformation into genes based on Entrez Genes/Unigenes allowed the identification of 44 and 44 genes in S9 cells, and 266 and 318 genes in 16HBE14o- cells that appeared up-regulated and down-regulated under influence of rHla, respectively. From these, 19 genes are transcribed at higher levels in both S9 and 16HBE14o- cells at comparable rates. For example, mRNAs of NR4A members 1 and 2, *FOS*, *IL6*, *DUSP1*, *EGR1* and *JUN* were observed in this group. 25 genes show decreased transcription levels in both cell lines, including *GPR21*, *CCL2*, *PMEPA1* and *SERPINE1*. The comparison of the transcriptome and proteome data revealed a number of direct correlations between mRNA and protein expression changes, e.g. down-regulation of plasminogen activator inhibitor *PAI1/SERPINE1* (proteome: -1.9, transcriptome: -2.1) and SDC4 (-2.4; -1.3), and up-regulation of Golgi phosphoprotein 3-like protein *GLP3L* (1.6; 2.5), observed for 16HBE14o- cells and histone H1.2 (*HIST1H1C*, -1.9; -1.5) in S9 cells.

We further examined genes with altered mRNA levels with regard to cellular function and activities. The observed up-regulation of *NR4A1*, *FOS*, *EGR1* and *JUN* specific mRNAs indicates the induction of an immediate early response mediated by rHla in S9 and 16HBE14o- cells (Fig. 4B). We performed a downstream effects analysis using the semi-automated pathway analysis software IPA in order to highlight biological trends in 16HBE14o- and S9 cells exposed to rHla. Considering the top five categories in the group *molecular and cellular functions* we identified cellular growth and proliferation, cellular development, and cell death as enriched in differentially expressed genes in 16HBE14o- and S9 cells similarly (Fig. 4C and S4 Table). Gene expression and cell cycle functional categories ranked in the top five for 16HBE14o- cells, whereas cellular movement and lipid metabolism ranked in the top five for S9 cells. Although affected categories in 16HBE14o- cells show overlap with S9 cells, and vice versa, we detected a marked difference between both types of cells when looking at the associated predicted increase or decrease of activity (Fig. 4D and S5 Table). The functional terms associated with cell proliferation (proliferation, tumorigenesis) appear as decreased in 16HBE14o- cells, whereas proliferation is predicted to be enhanced in S9 cells after rHla-treatment. Likewise, for 16HBE14o- cells cell death is predicted to be increased and cell viability is decreased, but in S9 cells cell viability is increased and apoptosis is decreased as predicted by the change of gene expression.

### Differential effects of EGFR and MAPK on transcriptional activation in rHla-treated 16HBE14o- cells or S9 cells

We hypothesized that rHla-mediated changes observed at the RNA level are preceded by activation changes in regulators upstream of gene expression that may match with observations of our phosphoproteome analysis. The correlation analysis of regulated genes to upstream regulator activity was limited to the molecule types ‘growth factor’, ‘kinase’ and ‘transmembrane receptor’ with the aim to specifically extract key factors of phosphorylation-dependent signaling. Since numerous upstream regulators with significant p-values (p≤0.05) were identified, we focused on the top 20 upstream regulators with highest p-values (S6 Table). Strikingly, EGFR and its agonist EGF were predicted as upstream regulators in rHla-treated S9 cells. As per activation z-score, EGF is significantly activated (p = 1.13E-18, z = 3.323) and EGFR shows activation by trend (p = 2.88e-16, z = 1.858). For 16HBE14o- cells in contrast, EGF (p = 1.86E-19, z = -1.033) and EGFR (p = 7.54E-18, z = -1.492) appeared inactivated after rHla-treatment by trend. We observed a similar association between differentially expressed genes and the upstream regulators MAPK3/1. While no change of activity was indicated in S9 cells (p = 5.19E-14, z = 0.836), a clear inhibition was inferred from data for 16HBE14o- cells (p = 3.52E-14, z = -2.98).

### Alpha-toxin-mediated EGFR activation in S9 cells is associated with its resistant phenotype

To clarify if the elevated EGFR and MAPK activity in S9 cells is associated with its resistant phenotype, we inhibited EGFR and MAPK activity by co-treatment of cells with the EGFR-selective inhibitor tyrphostin AG1478 and the MAPK-activating kinase MAP2K1/2 (MEK1/2) with PD98059, respectively.

**Fig 5:**
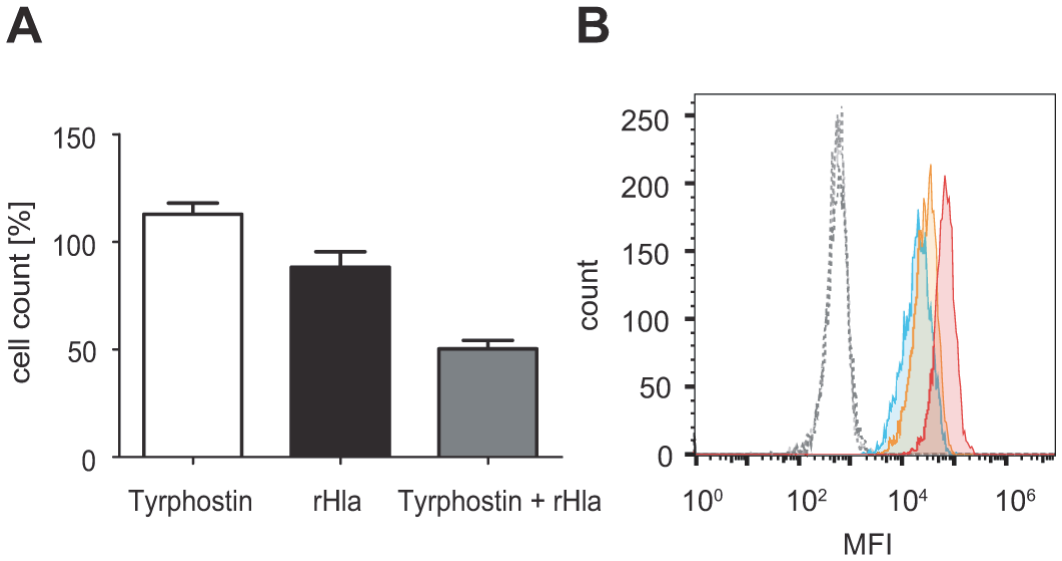
rHla-induced EGFR activation mediates resistance of S9 cells towards Hla. A. Cell counts of rHla-treated S9 cells co-treated with or without the EGFR-selective inhibitor tyrphostin AG1478. Graphs represent means ± SEM (n = 3). B. Quantification of EGFR surface expression in 16HBE14o- (red), A549 (blue) and S9 (orange) epithelial cells as analyzed by flow cytometry. The corresponding isotype control is indicated by a dashed line. MFI: mean fluorescence intensity.

Whereas S9 cell counts decreased by approximately 10% 6 h after addition of rHla in the control experiment, we monitored a significant decrease in survival of about 50% when cells were co-treated with rHla and tyrphostin AG1478 (Fig. 5A). This level of reduction in cell survival is comparable to 16HBE14o- cells treated with rHla for the same period of time. Interestingly, no significant effect on cell counts was observed when S9 cells were treated with the MAP2K1/2-selective inhibitor PD98059.

To further exclude an involvement of cell-type specific protein amounts of EGFR in EGFR and MAPK activity we quantified the cell surface-exposed EGFR fraction of 16HBE14o- and S9 cells as well as in a human alveolar cell line A549 by flow cytometry [45, 46]. The fraction of positively stained cells was in any case almost 100%, suggesting that all three cell lines are consistently equipped with surface exposed EGFR (Fig. 5B). EGFR-amounts were highest on 16HBE14o- cells followed by significantly reduced amounts on S9 and A549 cells at levels of 43% and 30%, respectively. The results therefore do not indicate a relation between EGFR content and EGFR activity.

### ADAM10 expression levels are different in three lines of airway epithelial cells and correlate with their rHla-sensitivities

**Fig 6:**
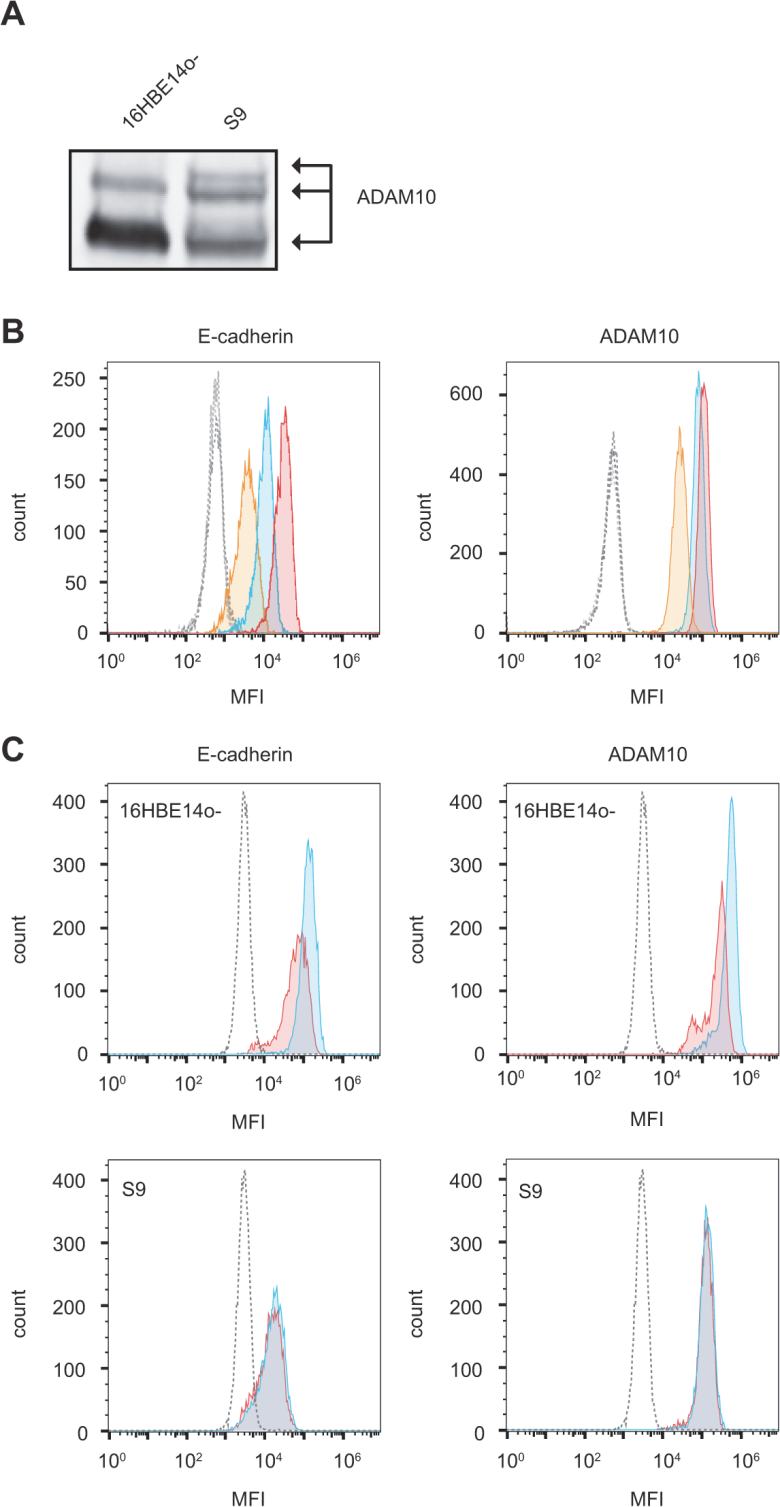
Involvement of surface proteins in Hla mediated cytotoxicity. **A.** Western blot analysis of ADAM10 expression in 16HBE14o- and S9 cells. Signals corresponding to mature and processed ADAM10 are indicated. B. Quantification of surface E-cadherin (left panel) and ADAM10 (right panel) in 16HBE14o- (red), A549 (blue) and S9 (orange) cells as analyzed by flow cytometry. Corresponding isotype controls are indicated as dashed shapes. C. Flow cytometry analysis of surface expression of E-cadherin (left panels) and ADAM10 (right panels) in 16HBE14o- (upper panels) and S9 (lower panels) cells after 2 h under control conditions (blue) or following 2 h treatment with rHla (red). The corresponding isotype control is indicated by a dashed line. MFI: mean fluorescence intensity.

Primarily based on experiments with human A549 alveolar cells, ADAM10 has recently been identified as a proteinaceous membrane-receptor for Hla which is involved in pore formation and mediation of toxic effects [9]. We therefore compared the level of ADAM10 in 16HBE14o-, S9 cells and A549 cells. Western blot analysis revealed a higher expression of ADAM10 in whole cell lysates of 16HBE14o- cells compared to S9 cells (Fig. 6A). Similar results were obtained for surface-exposed ADAM10 as analyzed by flow cytometry. S9 cells possess only 25% and A549 cells 80% of the surface-accessible ADAM10 content compared to 16HBE14o- cells (Fig. 6B, right panel). To characterize the role of ADAM10 in rHla-mediated responses in S9 and 16HBE14o- cells in more detail, we manipulated the level of ADAM10 by siRNA-mediated knockdown and studied the influence on cell viability and activation of specific kinases during rHla-treatment (S3 and S4 Figs.). Treatment with siRNAs resulted in efficient ADAM10 reduction in either cell line (S3A Fig.). Upon reduction of ADAM10 protein levels we observed a marked reversal of the deleterious effect of 6 h rHla-treatment on general metabolic condition of S9 cells (S3B Fig.).

Recently, activation of ADAM10 with subsequent cleavage of its substrate E-cadherin and concomitant dissolution of adherence junctions has been highlighted as a major mechanism of Hla-induced cytotoxicity [22, 47]. In line with this, we detected drastically higher levels of surface exposed E-cadherin in the highly Hla-sensitive 16HBE14o- cells compared to S9 cells exhibiting 7-fold decreased membrane located E-cadherin content (Fig. 6B, left panel). In addition, the numbers of positive cells suggest that a sub-population of roughly 20% did not carry E-cadherin at the cell surface whereas 16HBE14o- cells were homogenously decorated with E-cadherin. When challenged with rHla for 2 h, the amount of cell surface located E-cadherin dropped by approximately 50% in 16HBE14o- but not in S9 cells whereas the E-cadherin content was unaffected (Fig. 6C, left panel). Interestingly, a similar decline was observed for ADAM10 rHla-treated 16HBE14o-, but not in S9 cells (Fig. 6C, right panel).

If ADAM10 would be responsible for meditating rHla-triggered intracellular signaling, we would expect that suppression of ADAM10 expression results in inertness of intracellular signaling pathways to rHla-treatment of cells. To exemplify this, we suppressed ADAM10 expression using siRNAs and measured rHla-mediated changes in the phosphorylation levels of Y141 in PAK2 or Y576 in FAK, respectively. As indicated by the data reported in S4 Fig., we did not observe negative effects of suppression of ADAM10 expression on rHla-mediated changes in phosphorylation levels in PAK2 or FAK (S1 Fig.).

## Discussion

Healthy epithelia in the respiratory tract function as major barriers against airborne microorganisms including pathogenic *S*. *aureus* strains. One basis of defense is the mucociliary clearance that prevents the contact of bacteria to the cell surface. However, soluble toxins secreted by pathogenic bacteria may compromise epithelial cell function and may enable bacterial colonization and initiation of infection. Staphylococcal pore-forming Hla has been detected in patients suffering *S*. *aureus* diseases [48, 49] and was identified as major virulence-associated factor, since vaccination against Hla protected animals from developing *S*. *aureus*-mediated pneumonia [1]. However, contrasting with numerous studies that aimed to enlighten selected Hla-mediated implications in different models, global characterizations of the host response during host cell interaction with *S*. *aureus* or its virulence factors are still scarce.

In this study, we have for the first time used a multi-omics approach involving phosphoproteomics, proteomics and transcriptomics in combination with functional analyses to characterize phosphorylation-based signaling in human airway epithelial cells exposed to staphylococcal pore-forming cytotoxin Hla. This approach has identified that Hla drastically impacts the phospho-signaling of the bronchial epithelial cell lines 16HBE14o- and S9 after two hours of rHla-treatment. Notably, none of the highlighted proteins with altered phosphorylation showed significant changes at the protein level, confirming that Hla primarily impacts cellular signal transduction during the early phase. However, rHla exhibits a strong effect on cell layer integrity and survival in 16HBE14o- cells while for S9 cells no or only a transient impact was observed ([18, 25], this study). Led by the contrasting resistance phenotypes of both airway epithelia models, we hypothesized that characteristic differences at the level of signaling and gene expression may exist that discriminate the sensitivity towards Hla in a cell-type specific manner. Hla-induced activation of the EGF-receptor was thereby identified as the primary event leading to rHla-resistance in S9 cells. In contrast, rHla-treatment of 16HBE14o- cells was observed to significantly decrease phosphorylation at EGFR-sites which is accompanied by decreased down-stream activity. Furthermore, the different signaling properties of EGFR in S9 and 16HBE14o- cells were found to be implicated in the regulation of distinct gene expression patterns at the level of RNA.

In both model systems, signaling pathways associated with cell anchorages and actin cytoskeleton were identified to be affected following rHla-treatment. In particular, several protein kinases were observed with similar alterations in activity following rHla-treatment. For example, the p21-activated protein kinases (PAKs) 2 and 4 were found to be less phosphorylated at kinase activation sites in 16HBE14o- and S9 cells. PAKs are effectors of the Rho family GTPases Rac and Cdc42 that regulate actin cytoskeletal remodeling [50]. Decreased PAK activity mediates reduced phosphorylation of cofilin at S3, which eventually leads to a destabilization of actin filaments [51]. Consistently with the observed regulation of PAK activity, S3 of cofilin appeared to be less phosphorylated after rHla-treatment according to our phosphoproteomic analysis. Further regulators down-stream of Rac/Cdc42 that had been detected with reduced phosphorylation include IRSp53 (both cell models) as well as N-WASP (16HBE14o- cells). Both are reported to stimulate the Arp2/3 complex to induce rapid actin polymerization [52]. The tyrosine kinases FAK and Src are major components of the signaling complex in focal adhesions, which link the actin cytoskeleton via integrins to the extracellular matrix. The cooperative action of FAK and Src is thereby critical in the dynamic assembly and disassembly of focal adhesions [53, 54]. The monitored increased phosphorylation levels of FAK and Src upon treatment of cells with rHla, primarily at Y397/576 and Y419, respectively, is indicative for the activation of their down-stream kinase activities [55, 56]. Accordingly, also paxillin—a major FAK/Src substrate within the focal adhesions [53, 57] was found to display increased phosphorylation at Y118. Our results are in line with Hermann et al. [25] who demonstrated activation of FAK and increased phosphorylation of paxillin in 16HBE14o- and primary human nasal cells upon treatment with rHla.

We also identified multiple components assigned to tight junctions and adherence junctions having altered phosphorylation levels following rHla-treatment. Both cell-types showed decreased phosphorylation at Y280 of the junctional adhesion molecule JAM1, a recruitment point for the SH2-domain of the tyrosine kinase CSK [58]—a negative regulator of Src-family kinases [59, 60]. Treatment of cells with rHla also results in decreased phosphorylation within the tight junction protein ZO1 at S166/168, 617 and 912. Phosphoserine-166 is known to be implicated in lamellae formation in migrating cells via formation of a ZO1/alpha(5) integrin complex [61]. In addition, several actin binding adaptors were found with decreased phosphorylation, such as Afadin—a target of Rap1/Ras GTPases [62, 63]—and Protein 4.1 [64].

The observed rHla-mediated alterations in the signaling networks suggest a disturbance of the dynamics and/or stability of cellular structrres associated with cell-cell-contacts, cell-matrix-contacts and the cytoskeleton that may ultimately result in irreversible cell damage. In agreement with the cytotoxicity assays, immunofluorescence microscopy indicates a distinct disturbance of cell adhesion sites and the actin cytoskeleton which is accompanied by loosening of cell-cell contacts and appearance of paracellular gaps almost exclusively in 16HBE14o- cells but only transiently in S9 cells ([25], this study). Differing activities of individual key regulators of cellular signal transduction might determine the diverging behavior of 16HBE14o- and S9 cells in response to rHla. To study this more closely, we focused on kinases with differential regulation under rHla. The mitogen-activated protein kinases MAPK1 and MAPK3 and the epidermal growth factor receptor EGFR showed opposite phosphorylation changes at kinase activation sites, i.e. increased activity in S9 and decreased down-stream activity in 16HBE14o- cells following rHla-treatment. Activation of EGFR in 16HBE14o- cells promotes epithelial repair in a wound model [65]. This effect is attenuated by the EGFR-specific inhibitor tyrphostin AG1478 that impairs wound closure [66], indicating a vital role for EGFR activation in epithelial integrity. In our study, inhibition of the EGFR in S9 cells using tyrphostin AG1478 resulted in sensitization of S9 cells towards rHla rendering the S9 cells almost as sensitive to rHla as 16HBE14o- cells. Our findings strongly imply a critical link between Hla-induced activation of EGFR and resistance towards Hla-mediated toxicity in general. Recently, Haugwitz and coworkers reported a Hla-mediated mitogenic effect in HaCaT cells that could be attributed to Hla-induced EGFR activation [19]. Although an EGFR-dependent regulation of MAPK3/1 via the classical EGFR-Ras-Raf-MAP2K1/2-MAPK3/1 pathway seems likely, prevention of MAPK3/1 phosphorylation using the MAP2K1/2-specific inhibitor PD98059 did not result in sensitization of S9 cells towards rHla. Noteworthy, EGFR-independent signaling events also appear to be involved in the rHla-mediated activation of MAPK in S9 cells, because rHla-induced phosphorylation of MAPK3/1 still occurred in EGFR- as well as MAP2K1/2-inhibited cells, although to a lesser extent (S5 Fig.). We therefore suggest that the cell protective effect of rHla-mediated EGFR activation is either completely independent of MAPK3/1 down-stream activity or alternative activation pathways may act in cooperation. For the latter scenario, MAP2K1/2-independent MAPK activation was demonstrated via the protein kinase C and/or phosphatidylinositol-3-kinase pathway in different cell types [67–69]. However, the exact mechanisms of cell type-specific activation or deactivation of EGFR and MAPK by rHla remains elusive at this stage and needs to be addressed in future studies. Novel hypotheses in this regard could also be derived from our proteomic results, because several proteins with functions in cellular signal transduction were found to be altered in a cell type-specific manner. For example, the receptor-type tyrosine-protein phosphatase eta (PTPRJ) was depleted in rHla-treated S9 cells. PTPRJ negatively regulates EGFR and MAPK3/1 by dephosphorylation [70, 71] and its depletion may be involved in hyperactivation of EGFR and MAPK during rHla-treatment.

The observations that S9 cells are much better able to cope with rHla-treatment compared with 16HBE14o- cells was also evident by the results of our transcript profiling experiments. Treatment of 16HBE14o- cells with rHla activated several genes whose products are associated with induction of apoptosis and cell death while genes whose products are associated with cell proliferation, cell differentiation and cellular adhesion were found to be down-regulated. Genes whose products mediate cell proliferation, cell differentiation and survival, on the other hand, were activated by rHla-treatment in S9 cells. Consistent with the change of the phosphorylation state of the EGFR, our upstream regulator analysis based on different gene expression changes at transcriptional level indicated a potential activation of EGF and EGFR in S9 but inactivation in 16HBE14o- cells under rHla. These findings suggest that the divergence in gene expression in 16HBE14o- and S9 cells contributes to the differing tolerance of both cell types to rHla and is, at least in part, dependent on the modulation of EGFR-activity.

Another potential cellular feature that determines the sensitivity to Hla are different amounts of cell surface-exposed ADAM10 which functions as a receptor for Hla. Our results coincide with the notion that higher expression levels of ADAM10 may correlate with a higher degree of sensitivity to Hla [9], as 16HBE14o- cells show substantially higher levels of ADAM10 compared with S9 cells. Furthermore, interaction of Hla with ADAM10 results in activation of the latter and the subsequent destruction of cellular adhesion molecules, e.g. E-cadherin [23]. In line with this, the abundance of surface-exposed E-cadherin decreased dramatically in rHla-treated 16HBE14o- cells, but virtually not in S9 cells under the same conditions. Knockdown of ADAM10 significantly improved the 16HBE14o- phenotype, strongly indicating that expression of ADAM10/E-cadherin is a major determinant for the Hla-induced breakup of the cellular layer via ADAM10-dependent cleavage of E-cadherin. However, ADAM10 is likely not involved in mediation of the rHla-induced changes in the phosphoproteome in general, because phosphorylation and de-phosphorylation of FAK and PAK, respectively, is not changed in rHla-treated ADAM10 knockdown cells in both cell models.

Our data provide mechanistic insights into Hla-induced signaling in host cells and into the subsequent disruption of the epithelial cell layer. From our multi-omics approach, we propose that EGFR and downstream EGFR-targets represent the key hubs that determine the susceptibility to Hla in a cell-type specific manner. Central to the ability of *S*. *aureus* to cause disease in multiple body tissues with diverse clinical manifestations is the multitude of virulence factors that are produced in a regulated manner. The recent identification of cellular receptors for many virulence factors provides new insights into their cell type-specific action. The widely expressed Hla-receptor ADAM10 is thereby implicated in injury of host epithelium, endothelium and immune cells [3]. The effect on innate immune cells likely contributes to tissue-specific patterning of host outcome of infection [72, 73]. Regarding the cell type-specific effects of Hla, observations from this and our recent metabolomic study highlight additional aspects. We provided evidence that Hla has a significant impact on the metabolome of bronchial epithelial cells [18]. Hla-treated S9 cells thereby showed increased glycolytic rates that are likely advantageous by counteracting the Hla-mediated drop in the energy state. The observation that Hla-mediated alteration of activity of the surface receptor EGFR can be implicated in the level of resistance adds an additional layer of complexity and indicates that besides ADAM10 other molecules alone or in synergy shape the cellular responses to Hla. In the case of the EGFR, its rather prominent expression on epithelial cells renders the importance of the consideration of cell type-specific molecular features in further investigations on Hla-associated cellular responses especially in the context of the collective manifestation of all affects within the tissue microenvironment.

## Supporting Information

S1 Fig Verification of results obtained by phosphoproteomics using Western blot analyses. Western blot analyses of protein extracts derived from mock treated (-) or rHla-treated (+) 16HBE14o- and S9 cells after indicated incubation periods using phosphorylation-site specific and corresponding pan antibodies.
(PDF)

S2 Fig Microscopic characterization of 16HBE14o- and S9 cells under control conditions or upon treatment with rHla for 2 h. A) Representative light micrographs. B) Fluorescence micrographs of cells stained for actin (orange, TRITC-conjugated phalloidin), nuclear DNA (blue, Hoechst 33342) and vinculin (green, FITC-conjugated anti-Vinculin antibody). C) Fluorescence microscopy analysis of cells stained for E-cadherin (orange, PE-conjugated anti-E-cadherin antibody) and nuclear DNA (blue, Hoechst 33342).
(PDF)

S3 Fig Evaluation of ADAM10 depletion by Western blot analyses and involvement of ADAM10 in general cell fitness. A) Representative Western blot analyses for evaluation of siRNA-mediated ADAM10 knockdown efficiency in 16HBE14o- and S9 cells. Beta-actin was used as loading control. B) General metabolic activity determined by a resazurin-based assay of 16HBE14o- cells without and with siRNA-mediated ADAM10 knockdown in the absence or presence of rHla for 24 h.
(PDF)

S4 Fig Impact of ADAM10 depletion on Hla triggered (de)phosphorylation of FAK and PAK. Western blot analyses of activation sites of FAK (pY576) and PAK2 (pY141) of 16HBE14o- and S9 cells transfected with scrambled siRNA (control) or siRNAs targeting ADAM10 in the presence of rHla or mock control for 2 h.
(PDF)

S5 Fig Western blot analyses of Hla mediated MAPK1/3 activation in the presence of EGFR- and MAP2K1/2-specific inhibitors. Western blot analyses of MAPK1/3 activation site pT202/pY204 in S9 cells following 6 h rHla-treatment in the presence or absence of 10 μM EGFR-selective inhibitor tyrphostin AG1478 and 10 μM MAP2K1/2 inhibitor PD98059.
(PDF)

S1 Table SILAC-ratios of quantified phosphopeptides and phosphosites of rHla-treated 16HBE14o- and S9 cells vs. mock-treated cells. (XLSX)

S2 Table SILAC-ratios of quantified proteins of rHla-treated 16HBE14o- and S9 cells vs. mock-treated cells. (XLSX)

S3 Table Transcriptomic data of rHla-treated 16HBE14o- and S9 cells and mock-treated cells. (XLSX)

S4 Table Down-stream effect analysis of transcriptomic data obtained from rHla-treated 16HBE14o- and S9 cells. (XLS)

S5 Table Activation state prediction from transcriptome down-stream analysis. (XLSX)

S6 Table Up-stream regulator analysis of transcriptomic data obtained from rHla-treated 16HBE14o- and S9 cells. (XLS)
