# Supplementary figures and images for: A Multi-Omics Approach Identifies Key Hubs Associated with Cell Type-Specific Responses of Airway Epithelial Cells to Staphylococcal Alpha-Toxin
Source: PLoS One. 2015 Mar 27;10(3):e0122089. doi: 10.1371/journal.pone.0122089 (PMC4376684; doi:10.1371/journal.pone.0122089)

Supplementary Figure 1

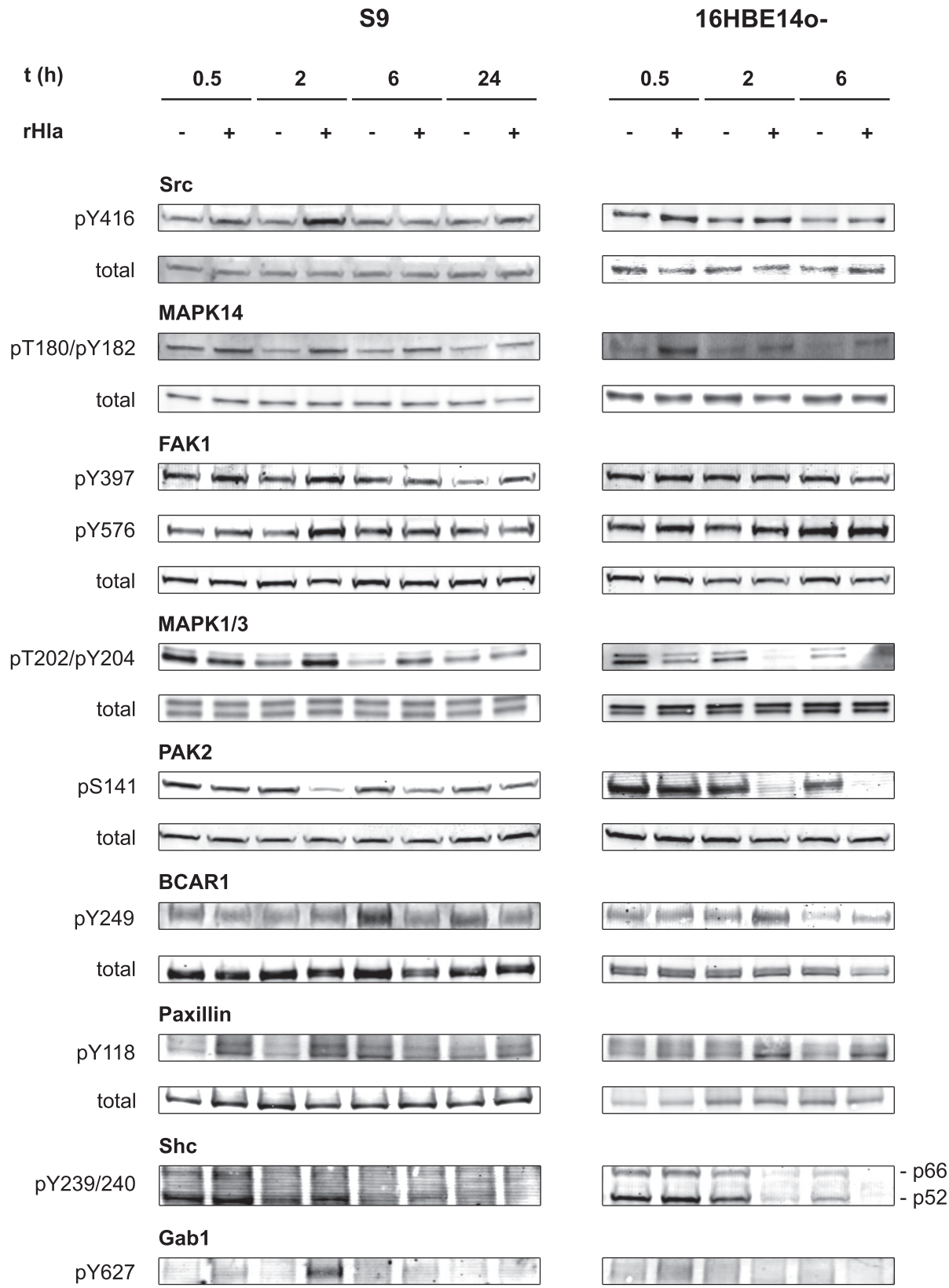

- p66  
- p52

Supplement: S1 Fig — Western blot analyses of protein extracts derived from mock treated (-) or rHla-treated (+) 16HBE14o- and S9 cells after indicated incubation periods using phosphorylation-site specific and corresponding pan antibodies. (PDF) [file pone.0122089.s001.pdf]

Supplementary Figure 2

16HBE14o-  
control

2h rHla treatment

S9  
control

2h rHla treatment

A

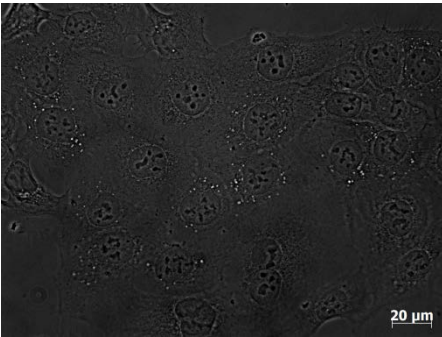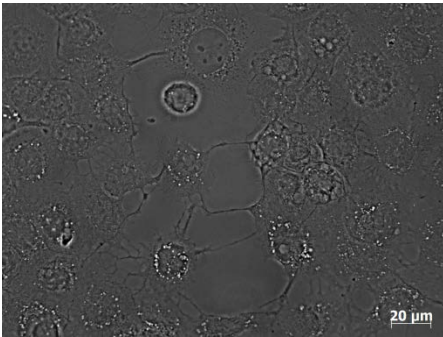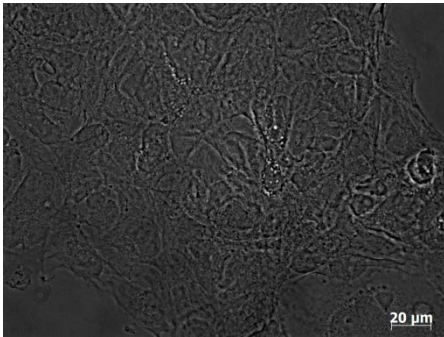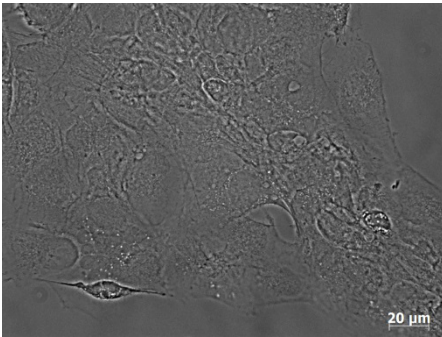

B

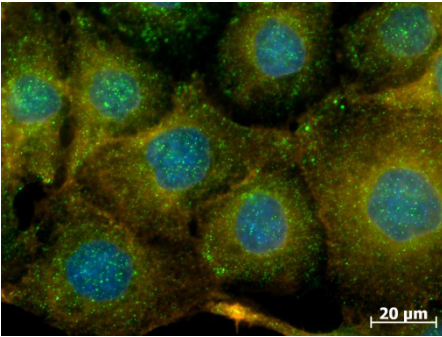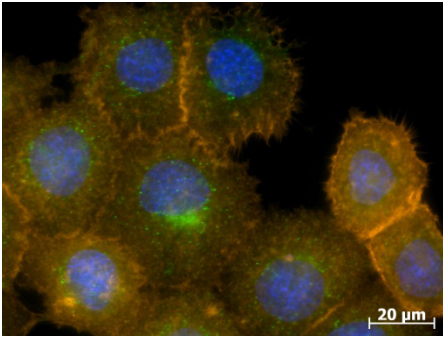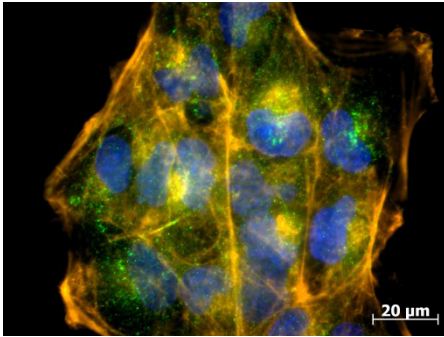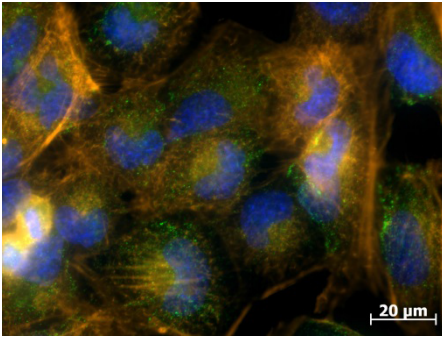

C

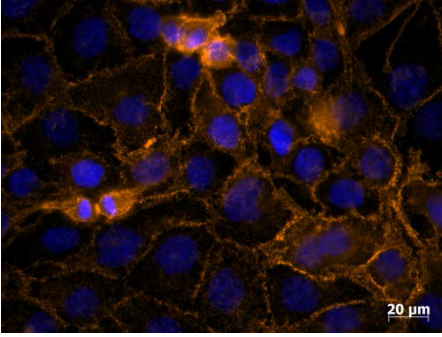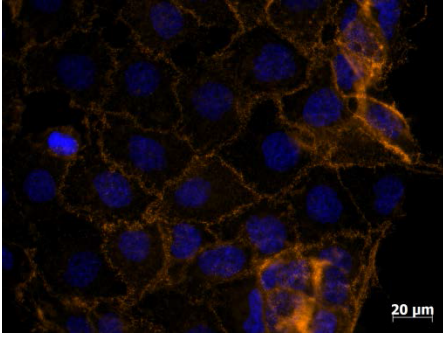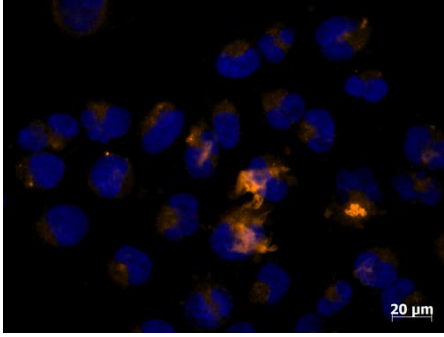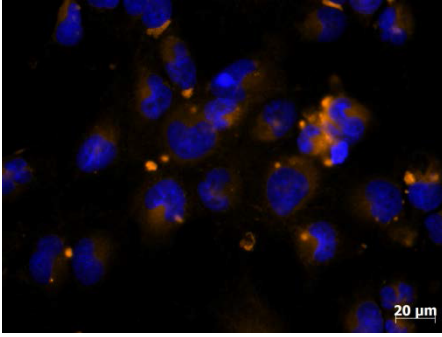

Supplement: S2 Fig — A) Representative light micrographs. B) Fluorescence micrographs of cells stained for actin (orange, TRITC-conjugated phalloidin), nuclear DNA (blue, Hoechst 33342) and vinculin (green, FITC-conjugated anti-Vinculin antibody). C) Fluorescence microscopy analysis of cells stained for E-cadherin (orange, PE-conjugated anti-E-cadherin antibody) and nuclear DNA (blue, Hoechst 33342). (PDF) [file pone.0122089.s002.pdf]

Supplementary Figure 3

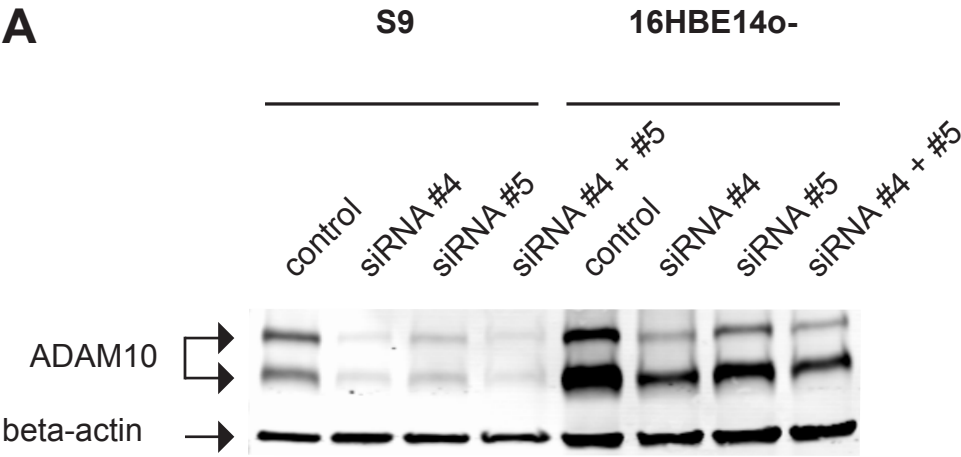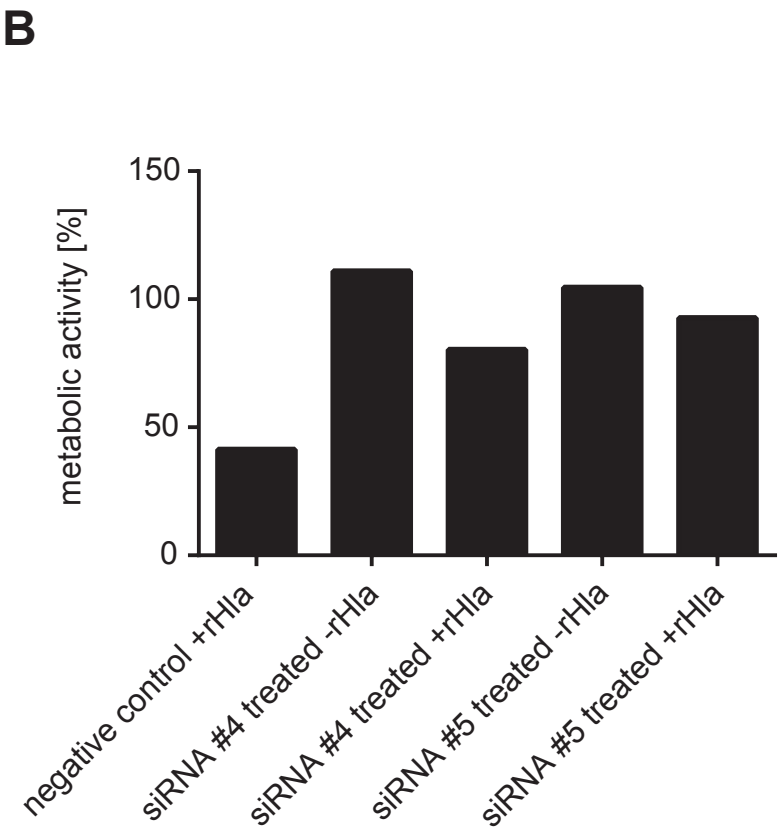

Supplement: S3 Fig — A) Representative Western blot analyses for evaluation of siRNA-mediated ADAM10 knockdown efficiency in 16HBE14o- and S9 cells. Beta-actin was used as loading control. B) General metabolic activity determined by a resazurin-based assay of 16HBE14o- cells without and with siRNA-mediated ADAM10 knockdown in the absence or presence of rHla for 24 h. (PDF) [file pone.0122089.s003.pdf]

Supplementary Figure 4

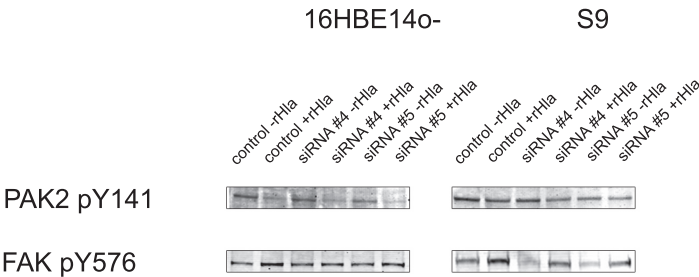

Supplement: S4 Fig — Western blot analyses of activation sites of FAK (pY576) and PAK2 (pY141) of 16HBE14o- and S9 cells transfected with scrambled siRNA (control) or siRNAs targeting ADAM10 in the presence of rHla or mock control for 2 h. (PDF) [file pone.0122089.s004.pdf]

Supplementary Figure 5

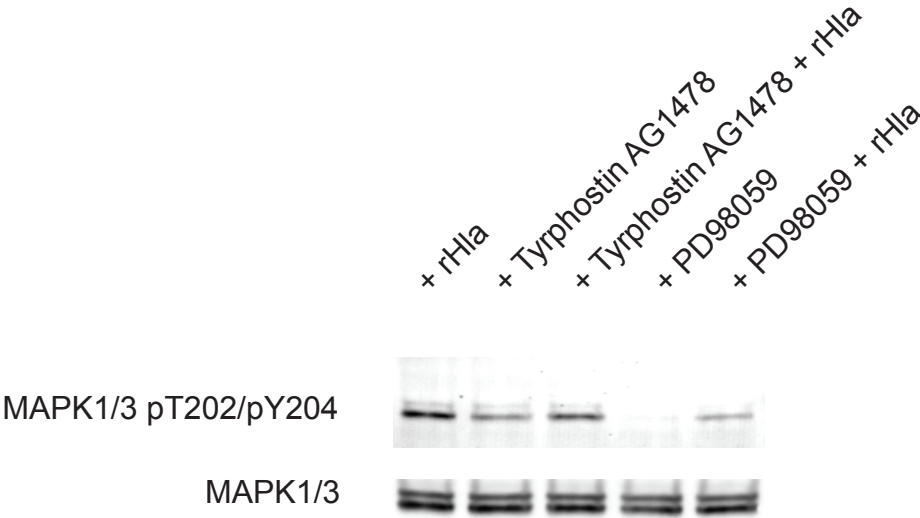

Supplement: S5 Fig — Western blot analyses of MAPK1/3 activation site pT202/pY204 in S9 cells following 6 h rHla-treatment in the presence or absence of 10 μM EGFR-selective inhibitor tyrphostin AG1478 and 10 μM MAP2K1/2 inhibitor PD98059. (PDF) [file pone.0122089.s005.pdf]
